# Synthesis, Spectroscopic, Thermal and Catalytic Properties of Four New Metal (II) Complexes with Selected N- and O-Donor Ligands

**DOI:** 10.3390/ma13143217

**Published:** 2020-07-20

**Authors:** Agnieszka Czylkowska, Bartłomiej Rogalewicz, Anita Raducka, Natalia Błaszczyk, Tomasz Maniecki, Kinga Wieczorek, Paweł Mierczyński

**Affiliations:** Institute of General and Ecological Chemistry, Faculty of Chemistry, Lodz University of Technology, Zeromskiego 116, 90-924 Lodz, Poland; 211150@edu.p.lodz.pl (B.R.); anita.raducka@dokt.p.lodz.pl (A.R.); natalia.blaszczyk@dokt.p.lodz.pl (N.B.); tomasz.maniecki@p.lodz.pl (T.M.); kinga.wieczorek@dokt.p.lodz.pl (K.W.); pawel.mierczynski@p.lodz.pl (P.M.)

**Keywords:** metal (II) complexes, imidazole, acetates, XRD, TG-DTG, DSC, TG-FTIR, catalyst, styrene oxidation

## Abstract

Four solid compounds with formulae: Co(OAc)_2_(Im)·H_2_O (I), Ni(OAc)_2_(Im)_1.5_·2H_2_O (II), Cu_2_(OAc)_4_(Im) (III) and Zn(OAc)_2_(Im)·H_2_O (IV) (where: Im = 1*H*-Imidazole) were prepared and characterized by chemical and elemental analysis, powder X-ray diffraction patterns and FTIR spectroscopy. Catalytic properties of each complex for styrene oxidation reaction were investigated. Furthermore, thermal properties of compounds were studied using the TG-DTG and DSC techniques under dry air atmosphere. Additionally, volatile thermal decomposition and fragmentation products were also investigated using the TG-FTIR spectra in air.

## 1. Introduction

Complexes are chemical compounds which can be characterized by various functions induced by ligands incorporated into their structure. One of the most widely used ligands in complexes’ structures is imidazole. Complexes containing an imidazole ring in their structure are widespread in natural environment and many of them display important biological functions. This N-Donor ligand itself is a well-known heterocyclic aromatic compound which is commonly used as a component of medicines. Many of its derivatives, such as alkaloids and antifungal drugs, have biological functions. Imidazoles are a group of antifungal agents that was introduced in the 1980s, which can be divided into two groups: diazoles—containing two nitrogen atoms in the five-membered azole ring [1,2,3,4,5,6,7,8]. The second ligand has carboxylate group which can create different types of binding with metal ions [9,10]. It often acts as bidentate ligand, through electron pairs of two oxygen atoms in its molecule [11,12,13]. In addition, it is used in various industries and in chemical synthesis for the production of other important chemicals. This well-known building block is widely used in cosmetics as a pH regulator. It is also used in food production as a preservative: it protects food against development of bacteria and fungi, thus prolonging their durability. It is also used for dyeing fabrics in the textile industry [14,15]. Coordination compounds of metal ions with both organic ligands are an interesting and promising field of research. In literature we can find similar compounds to those we obtained, however, with different stoichiometric ratios. They have been synthesized and structurally described [16,17,18,19,20,21,22,23]; some of them form dimeric metal complexes [24,25,26,27] or polymeric structures [28,29]. Such variety of possible interactions between metals and ligands draws attention to further investigations of these compounds, for this type of compounds thermal and catalytic properties have not been studied yet. The material in this paper fills an existing gap in the literature on these compounds. Here, we present synthesis and investigation of physical and chemical properties of new solid Co (II), Ni (II), Cu (II) and Zn (II) complexes with title ligands. After establishing the composition, we thoroughly investigated their thermal properties. It is an important issue, taking into consideration their possible future applications. All of them are stable at room temperature and, when heated, decompose to neutral metal oxides. One of the possible future applications of these compounds is catalysis. Their catalytic properties have been tested for styrene oxidation reaction with promising results. Besides satisfactory conversion, these compounds can also be described as stable and relatively simple structures. Oxidation reaction of aromatic compounds like styrene draws interest not only from researchers but also from industry. Oxidation reaction speed largely depends on the nature of these compounds, their molecular weight and steric configuration. Usually oxidation of styrene is carried out in the presence of peracids but this process leads to undesirable products. To overcome these limitations, an ecological oxidizer (H_2_O_2_) is used. However, the most important issue is the choice of catalyst that allows for reasonable conversion of the compounds. Therefore, Co (II), Ni (II), Cu (II) and Zn (II) complexes have been tested as catalysts in the styrene oxidation reaction [30,31].

## 2. Materials and Methods 

### 2.1. Materials, Synthesis and Analysis 

Imidazole, acetic acid, CoCO_3_, NiCO_3_·2Ni(OH)_2_, CuCO_3_·Cu(OH)_2_ and ZnCO_3_ from Sigma Aldrich (Poland) were used. Water solutions of metal (II) acetates were prepared by adding 2 mol·L^−1^ acetic acid to metal (II) carbonates or metal (II) carbonate hydroxides. The reactions were carried out for 24 h at room temperature (25 °C). After that time, the undissolved precipitates were filtered off. Obtained metal (II) acetates solutions were further analyzed: after appropriate dilution, contents of metal (II) ions were determined by AAS technique. Standard solutions Merck (1000 mg/L, Darmstadt, Germany) were used for preparation of calibration curves. For analysis, distilled water with electrical conductivity 0.05 µS was applied (water deionizer system Polwater).

**Figure 1:**
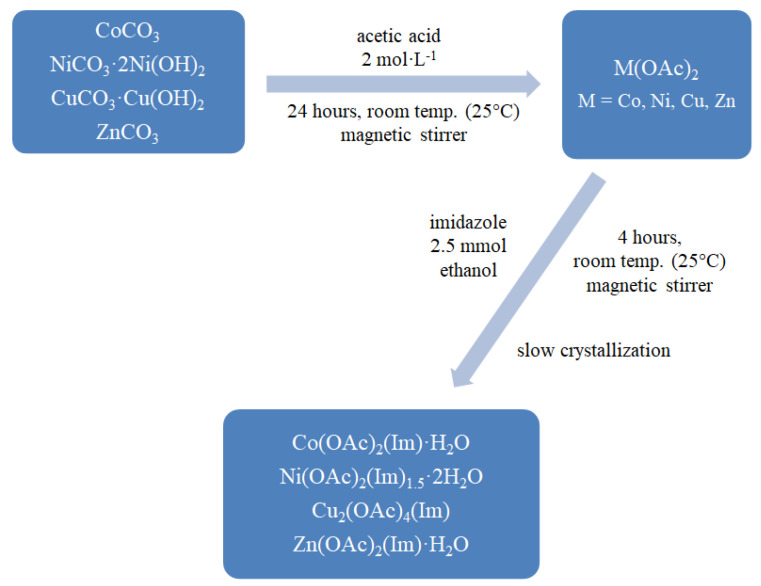
Synthesis path for mixed-ligand complexes.

The mixed-ligand complexes were prepared by mixing 2.5 mmol of imidazole in 96% v/v ethanol with freshly obtained water solutions of 2.5 mmol metal (II) acetates. Reaction mixtures were then stirred on a magnetic stirrer for 4 h. The synthesis was carried out at room temperature (pH = 5–6). After a few days, compounds were crystallized. Products were filtered off and dried in air at room temperature. Synthesis is schematically presented on Figure 1 and described by reactions below.

MCO3+2HOAc →24 h,room temp. M(OAc)2+CO2+H2O, where M (II)=Co, ZnCuCO3·Cu(OH)2+4HOAc →24 h, room temp. 2Cu(OAc)2+CO2+2H2ONiCO3·2Ni(OH)2+6HOAc →24 h, room temp. 3Ni(OAc)2+CO2+3H2OM(OAc)2·H2O+Im →4 h, room temp. M(OAc)2(Im)·H2O ↓, where M (II)=Co, Zn2Cu(OAc)2+Im →4 h, room temp. Cu2(OAc)4(Im) ↓Ni(OAc)2·2H2O+1.5Im →4 h, room temp. Ni(OAc)2(Im)1.5·2H2O ↓

Samples of complexes (about 20 mg) were digested in a mixture of concentrated 36% HCl (1 mL) and 65% HNO_3_ (6 mL) and the contents of metals were determined using the same methodology as for the metal (II) acetates solutions.

The contents of C, H and N in prepared compounds were determined by a Vario micro company Elementar Analysensysteme GmbH (Langenselbold, Germany)

The catalytic activity tests were carried out in liquid phase, product analysis was done using GC-chromatograph equipped with FID detector (HP 5890) (Hewlett Packard Corporation, Palo Alto, CA, USA). The catalytic liquid-phase oxidation reactions of styrene were used to study the catalytic activity of complexes. Reactions were carried out in a 50 mL round-bottom flask. The molar ratio of reagents was C_2_H_3_N:H_2_O_2_:C_8_H_8_ = 1:1:1. The mixture was stirred in a water bath at 60 °C and refluxed. The catalyst (0.01 g) was added after reaching this temperature. 

The analysis of styrene content was performed by gas chromatography equipped with a flame ionization detector (GD-FID, HP 5890, Hewlett Packard Corporation). The optimal parameters of method were as follows: separation column: ZB-FFAP capillary column (30 m × 0.25 mm × 0.25 µm); oven profile: 60 °C for 8 min to 150 °C/min for 4 min; injection temperature 225 °C; injection split: 11:8:1, 0.5 µL; detector temperature: 250 °C; carrier gas: helium 3.4 mL/min. Conversion degree was determined according to the following formula:

(1) $$\mathrm{Conversion}=\left[\frac{A_{0}-A_{2}}{A_{0}}\right]\times 100\%$$

A_0_ is the initial concentration of styrene in reactant mixture; A_2_ is the concentration of styrene after 2 h in reactant mixture.

### 2.2. Methods and Instruments

The contents of Co (II), Ni (II), Cu (II) and Zn (II) in acetates and solid complexes were determined by the F-AAS spectrometer (Analityk Jena, contraAA 300, Jena, Germany) with a continuum source of light and using air/acetylene flame (Analityk Jena, contraAA 300). Absorbances were measured at analytical spectral lines: 240.7 nm for Co (II), 232.0 for Ni (II), 324.7 nm for Cu (II) and 213.9 nm for Zn (II). Limits of quantification were 0.04 mg/L for Co (II) and Cu (II), 0.005 mg/L for Zn (II) and 0.09 mg/L for Ni (II). Solid samples were decomposed using the Anton Paar Multiwave 3000 (Graz, Austria) closed system instrument. Mineralization was carried out for 45 min at 240 °C under pressure 60 bar. FTIR spectra were recorded with an IRTracer-100 Schimadzu Spectrometer (3800–800 cm^−1^ with an accuracy of recording of 1 cm^−1^, Kyoto, Japan) using KBr pellets. Thermal properties of complexes in air were studied using STA 449 F1 Jupiter Netzsch (Selb, Germany) coupled with FTIR Tensor27 Bruker (Ettlingen, Germany) in the temperature range 25–1000 °C at a heating rate of 10 °C·min^−1^, in flowing dynamic air atmosphere v = 20 mL·min^−1^ using ceramic crucibles; as a reference material, ceramic crucibles were used. Room temperature powder X-ray diffraction patterns were collected using a PANalytical X’Pert Pro MPD diffractometer (PANalytical, Almelo, The Netherlands) in the Bragg–Brentano reflection geometry. Copper CuK_α_ radiation from a sealed tube was used. Data were collected in the 2θ range 5–90° with a step of 0.0167° and an exposure per step of 50 s. The samples were spun during data collection to minimize preferred orientation effects. A PANalytical X’Celerator detector based on Real Time Multiple Strip technology and capable of simultaneously measuring intensities in the 2θ range of 2.122° was used. 

## 3. Results

### 3.1. Elemental Analysis

As a result of the two-step synthesis, four new solid mixed-ligand complexes with the following formulas: Co(OAc)_2_(Im)·H_2_O (I), Ni(OAc)_2_(Im)_1.5_·2H_2_O (II), Cu_2_(OAc)_4_(Im) (III) and Zn(OAc)_2_(Im)·H_2_O (IV) were obtained. Table 1 presents results of the elemental and chemical analysis of investigated complexes. These compounds are stable in air in solid state. They do not change their stoichiometric composition.

### 3.2. X-ray Diffraction Data

**Figure 2:**
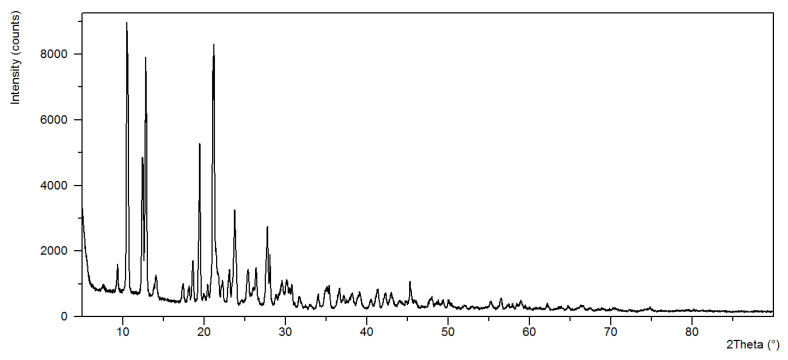
The diffraction pattern of Co(OAc)_2_(Im)·H_2_O.

**Figure 3:**
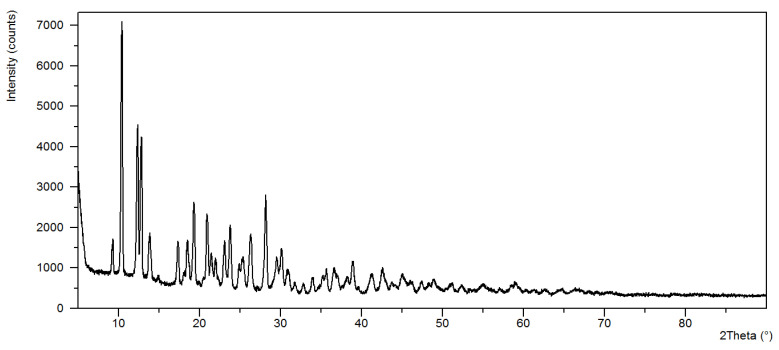
The diffraction pattern of Ni(OAc)_2_(Im)_1.5_·2H_2_O.

**Figure 4:**
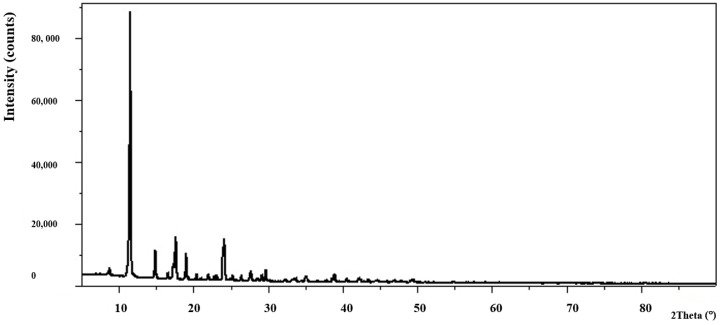
The diffraction pattern of Cu_2_(OAc)_4_(Im).

**Figure 5:**
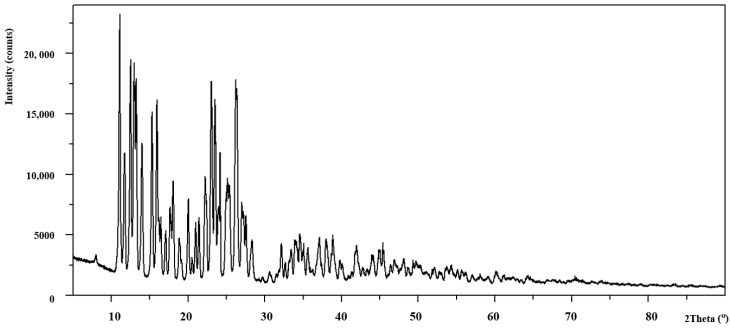
The diffraction pattern of Zn(OAc)_2_(Im)·H_2_O.

The analysis of power diffraction patterns of these compounds revealed that they are small crystalline (Figure 2, Figure 3, Figure 4 and Figure 5). Although Co (II) and Zn (II) complexes have the same composition, they are not isostructural compounds. Powder diffraction patterns of all complexes do not occur in Powder Diffraction File [32].

### 3.3. FTIR Spectra

**Figure 6:**
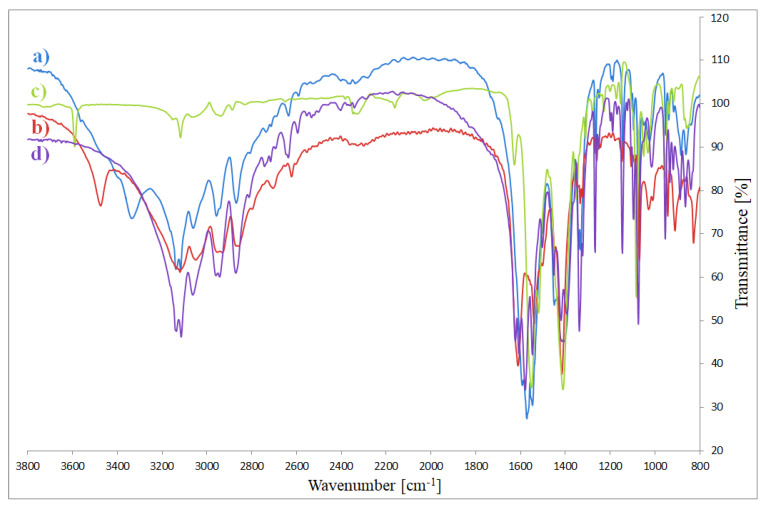
FTIR spectra of complexes: (**a**) Co(OAc)_2_(Im)·H_2_O, (**b**) Ni(OAc)_2_(Im)_1.5_·2H_2_O, (**c**) Cu_2_(OAc)_4_(Im), (**d**) Zn(OAc)_2_(Im)·H_2_O.

The fundamental vibration modes of imidazole, -COO groups and complexes are reported in Table 2 (Figure 6). During coordination with metal (II) ions, the vibration modes of free N and O-donors change. The fundamental stretching vibration modes *ν*(NH) and *ν*(CH) (in free ligand at 3124 cm^−1^) occur in the range 3143–3114 cm^−1^ in the complexes spectra. In the free ligand spectra, a strong band of around 1645 cm^−1^ is assigned to the vibration mode *ν*(C=N). For complexes this band is shifted to lower wavenumbers by 45–18 cm^−1^ compared to the spectra of free imidazole. In the absorption region of N-donor ligand appear also the modes: δ(CN), ν(CC) and ν(CN). In the free ligand, they are visible at 1540 cm^−1^. They are moved to the higher and lower frequencies as a result of coordination between metal ion and N-donor ligand. In the spectra of uncoordinated ligand there are also vibrations of δ(CN), π(CH) and δ(imidazole ring). In complexes, they are shifted in comparison to free imidazole. Changes of vibration modes *ν*(NH), *ν*(CH), *ν*(CN), δ(CN), ν(CC), δ(CN), π(CH) and δ(imidazole ring) of complexes in comparison to the spectra of free imidazole prove the existence of binding between imidazole and metal ions [33,34]. 

Acetate is the ligand which may coordinate in different ways. The way of coordination carboxylate group is described by spectroscopic criteria [35,36]. Nakamoto [35] and Alcock and co-authors [36] compared values of separation of asymmetric (ν_as_(COO)) and symmetric (ν_s_(COO)) frequencies of complexes with these bands for sodium salt of carboxylate ligand. The separation Δν = ν_as_(COO) − ν_s_(COO) characterizes the nature of metal carboxylate bond. When Δν_Na_ > Δν_complex_ carboxylate group is a bidentate chelating, in case of Δν_Na_ < Δν_complex_ it coordinates as monodentate ligand and for Δν_Na_ ≈ Δν_complex_ acts as bidentate-bridging donor [35,36].

In the spectra of complexes, there are also visible vibrations of asymmetric ν_as_(COO) and symmetric ν_s_(COO) modes for -COO groups from acetate ligands (Table 2). For Co (II) and Zn (II) compounds, Δν_complex_ are similar to sodium salt. It means that, in these complexes, -COO groups coordinate as bidentate-bridging ligands. The splitting of ν_s_(COO) bands existing in the spectra of Co (II) compound is probably caused by formation of non-completely equivalent bonds between metal (II) and carboxylate groups [37,38]. For the Cu (II) compound, Δν_complex_ is both similar and lower than for sodium salt. It means that in this complex the carboxylate groups act as bidentate-chelating and bidentate-bridging ligands. In the case of nickel (II) complex, ν_as_(COO) bands overlap with ν(CN) bands from N-donor ligand; therefore, it is difficult to determine its type of coordination.

### 3.4. Thermogravimetric Studies in Air

All four complexes are stable at room temperature. Their thermal decompositions have been studied in air using TG-DTG (Figure 7, Figure 8, Figure 9 and Figure 10) and DSC (Figure 11, Figure 12, Figure 13 and Figure 14) methods. Thermal decomposition data are exhibited in Table 3.

Thermolysis of Co(OAc)_2_(Im)·H_2_O begins at 80 °C. In the range of 80–125 °C, half of a water molecule is released with endothermic effect at 110 °C (mass loss: found. 4.0%; calc. 3.43%). Above 125 °C, a decomposition of another half of a water molecule and 0.5 of imidazole molecule is observed (mass loss: found. 17.0%; calc. 16.35%). They are accompanied by small endothermic peaks on the DSC curve. Between 225–410 °C, there is total destruction of organic ligands (mass loss: found. 49.5%; calc. 49.72%). It is confirmed by small endothermic and large exothermic effects on DSC curve. Process stops at 410 °C with Co_3_O_4_ as a final solid product of decomposition. 

Ni(OAc)_2_(Im)_1.5_·2H_2_O is stable to 50 °C. At this temperature, one and a half of water molecules are released. Mass loss is observed on TG curve: found. 8.0%; calc. 8.58% (endothermic effect at 105 °C). Next, further dehydration takes place. At 160 °C the decomposition of N-donor ligand starts (mass loss: found. 32.0%; calc. 32.43%), with a small endothermic peak on DSC curve. In the temperature range 375–440 °C, acetates decomposition occurs and NiO is formed. On DSC, there is one large broad exothermic effect.

Thermolysis of Cu_2_(OAc)_4_(Im) begins at 165 °C. It is associated with the release of imidazole molecule (mass loss: found. 16.0%; calc. 15.78%). An endothermic peak appears at 190 °C on the DSC curve. When the temperature rises (200–340 °C), partial decomposition of carboxylates takes place and an intermediate compound of the formula Cu_2_(OAc)_1.5_ is formed (mass loss: found. 34.0%; calc. 34.23%). It is connected with two exothermic effects on DSC curve at 250 and 260 °C. Next, total decomposition of organic ligands occurs. The curve shows presence of exo peak at 450 °C, which is associated with combustion of the remaining carbonization products. Horizontal mass level for pure CuO begins at 490 °C (found. 13.5%; calc. 13.12%). 

Thermal decomposition of Zn(OAc)_2_(Im)·H_2_O begins at 50 °C and is connected with releasing a half of water molecule (mass loss: found. 4.0%; calc. 3.34%) with endothermic peak at 80 °C. In the range of temperature 125–310 °C, further releasing of water and decomposition of an imidazole molecule occurs (mass loss: found. 28.0%; calc. 28.62%). These processes are accompanied by two endothermic effects. Above 310 °C, acetates are decomposed with endothermic and broad exothermic effects on DSC curve. The final solid product is pure ZnO.

### 3.5. TG-FTIR Studies in Air

**Figure 15:**
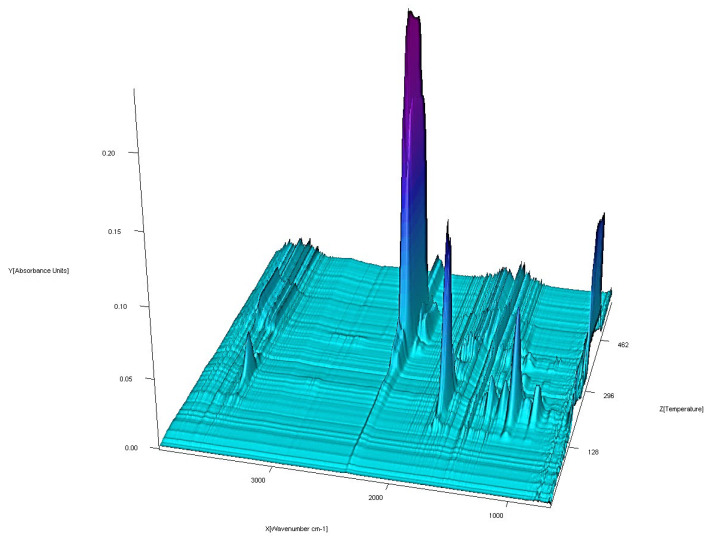
FTIR spectra of gaseous products produced during decomposition of Co(OAc)_2_(Im)·H_2_O complex in air.

**Figure 16:**
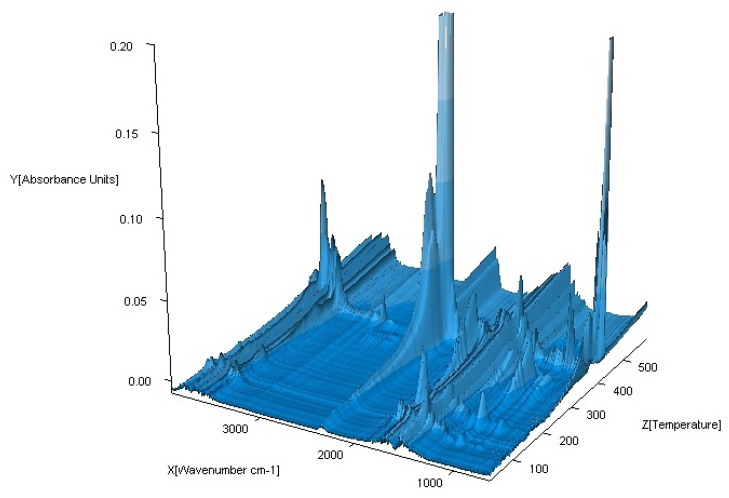
FTIR spectra of gaseous products produced during decomposition of Ni(OAc)_2_(Im)_1.5_·2H_2_O complex in air.

**Figure 17:**
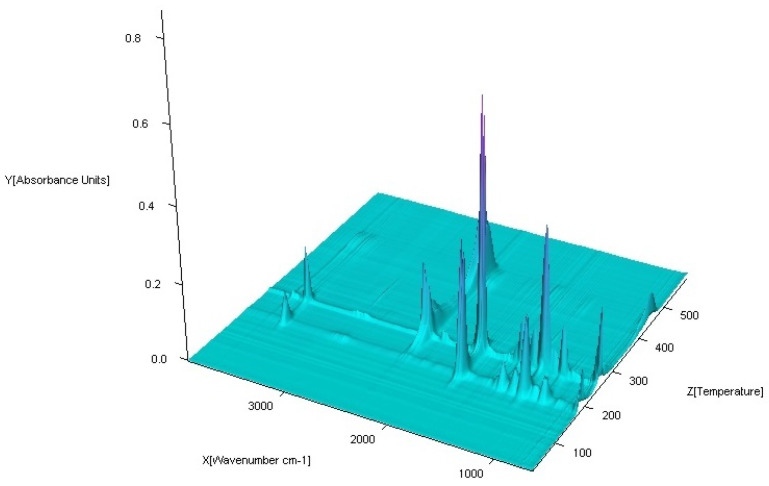
FTIR spectra of gaseous products produced during decomposition of Cu_2_(OAc)_4_(Im) complex in air.

**Figure 18:**
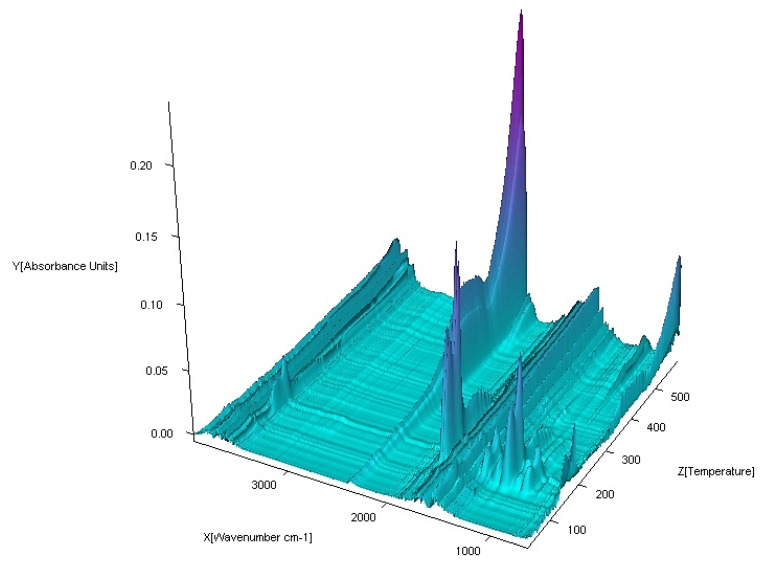
FTIR spectra of gaseous products produced during decomposition of Zn(OAc)_2_(Im)·H_2_O complex in air.

The coupled TG-FTIR techniques were carried out to analyze volatile thermal decomposition and fragmentation products (Figure 15, Figure 16, Figure 17 and Figure 18). Based on the analysis of TG-DTG and DSC curves, we know that for the Co (II), Ni (II) and Zn (II) complexes, the first step of decomposition is the elimination of 0.5 mol of water. Dehydration begins at 50 °C for the Ni (II) and Zn (II) complexes and at 80 °C for Co (II). When the temperature rises, for the Co (II) and Ni (II) compounds, total dehydration occurs and, additionally, only for Co (II) complex partial deamination take place. In the case of the Zn (II) complex, further loss of water goes hand in hand with total destruction of the N-donor ligand. Further heating causes a complete decomposition of organic ligands with the formation of the proper metal oxides.

The FTIR spectra of the gaseous products from the decomposition of Co (II), Ni (II) and Zn (II) compounds were similar. In low temperature of pyrolysis (about 100 °C for Ni (II), Zn (II) and 120 °C for Co (II)), there are bands of stretching and deformation vibrations of liberating water. These are in the ranges of 3800–3650 cm^−1^ and 1850–1300 cm^−1^, respectively. For the Cu (II) complex, gaseous products start to eliminate at about 200 °C. When the temperature rises, the bands of N-donor ligand are observed. These bands are corresponding to stretching vibrations of fragments CC and CN. These occur in the range of 1800–1500 cm^−1^. The destruction of imidazole also causes the appearance of vibrations bands β(CH) in plane in the range 1300–1200 cm^−1^ and γ(CH) out of plane at ca. 1000 cm^−1^. Decomposition of organic ligands is accompanied by the formation of water and carbon dioxide. The maximum release of water and carbon dioxide are during the thermodestruction of acetate ions. It has its impact on the spectra: the maximum CO_2_ evolution was recorded at about 360 °C for Co (II), 410 °C for Ni (II), 420 °C for Cu (II) and 560 °C for Zn (II) complex. The bands of the stretching and deformation molecules of CO_2_ are in the ranges of 2300–2250 cm^−1^ and 750–700 cm^−1^, respectively. At the same temperatures, maxima of water emission from decomposition and burning of organic ligands are observed. Further heating leads to a decrease of CO_2_ and H_2_O that proves the end of the thermal decomposition process. 

### 3.6. Activity Tests in Styrene Oxidation Reaction

**Figure 19:**
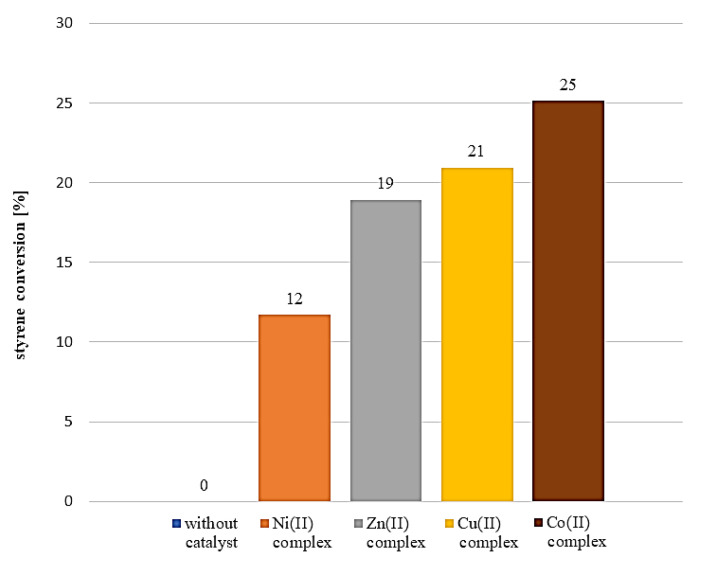
Percentage styrene conversion obtained with studied complexes used as catalysts.

All four complexes can be considered as promising highly effective catalysts for many processes which take place in liquid phase [39,40,41]. The catalytic efficiency was tested in styrene oxidation process in liquid phase (Figure 19). As the oxidizing agent, the hydrogen peroxide was used. The main product of that process was carbon dioxide; only small traces of benzaldehyde were detected in post reaction mixture. 

Catalytic effect is related to the presence of metal complexes. It is provided by comparison of experiments conducted without a catalyst and with catalysts. The lowest conversion (up to 12%) was observed for experiment carried out with the Ni (II) complex. For the Zn (II) and Cu (II) complexes, a higher conversion degree was observed: up to 19% and 21%, respectively. The highest conversion after 2 h of reaction was observed for the Co (II) complex: up to 25%. Another important determinant of utility of these compounds as catalysts is selectivity. In post reaction mixture only small traces of benzaldehyde were detected: all four complexes exhibit almost 100% selectivity towards carbon dioxide formation. The catalytic activity of metal complexes used in this experiment is associated with their structure and the type of metal atom and ligands. The difference in catalyst activity may be due to the difference in the acid-base properties of metals.

## 4. Conclusions

Four new mixed-ligand coordination compounds with formulae: Co(OAc)_2_(Im)·H_2_O, Ni(OAc)_2_(Im)_1.5_·2H_2_O, Cu_2_(OAc)_4_(Im) and Zn(OAc)_2_(Im)·H_2_O were synthesized and isolated. Changes observed in FTIR spectra of complexes indicate that the title ligands coordinate to metal (II) ions. The thermal analysis of all the complexes confirmed that they are stable at room temperature. During heating, they decompose progressively and several processes of decomposition are weakly separated from one to another. The most stable is Cu_2_(OAc)_4_(Im) (165 °C), since it does not contain any water molecule. When the temperature increases, only the Ni (II) complex loses total water content. In the case of Co (II) and Zn (II), this process is connected with partial and total deamination, respectively. Further heating leads to decomposition of acetates. The final solid products of pyrolysis are pure metal (II) oxides. The coupled TG-FTIR study made it possible to identify a number of gaseous species that formed and evolved during thermal decomposition of investigated complexes. The infrared spectra of volatile products recorded during heating of investigated compounds allow a better understanding of their thermal decomposition process. Emission of gaseous products in particular steps of thermal decomposition of obtained complexes corresponds with mass losses on TG curves. Performed thermal investigations are comprehensive and provide a precise view on this issue. These compounds also decompose in relatively low temperatures and final solid products of decomposition are simple inorganic oxides, which are easy to handle, store and reuse. It is especially important to take into consideration the possible use of these compounds in the future, for instance as catalysts. The catalytic activity of complexes was screened for styrene oxidation reaction. Studied complexes catalyze this reaction with satisfactory efficiency under moderate reaction conditions. All complexes exhibit almost 100% selectivity towards carbon dioxide formation. Only small traces of benzaldehyde were detected in post reaction mixture. Synthesized complex catalysts provide an efficient and safe approach to the oxidation of styrene to corresponding compounds using H_2_O_2_ as a mild oxidant.

## Figures and Tables

**Figure 7:**
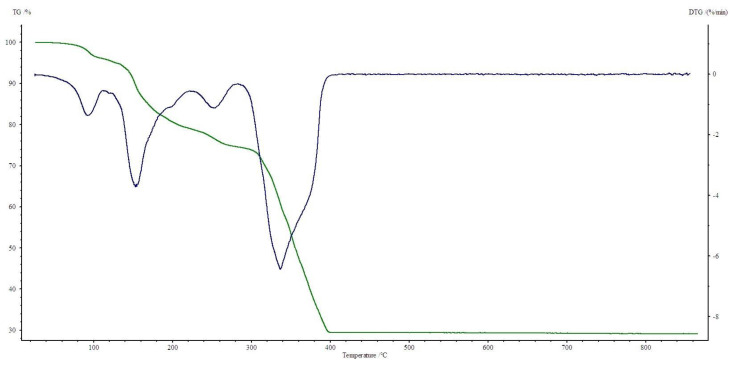
TG, DTG curves of Co(OAc)_2_(Im)·H_2_O complex in air.

**Figure 8:**
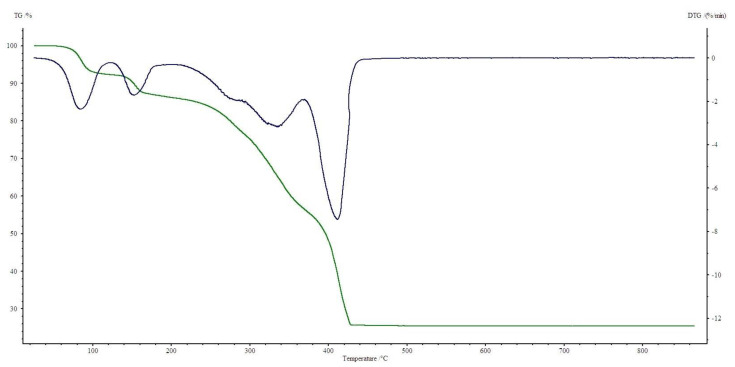
TG, DTG curves of Ni(OAc)_2_(Im)_1.5_·2H_2_O complex in air.

**Figure 9:**
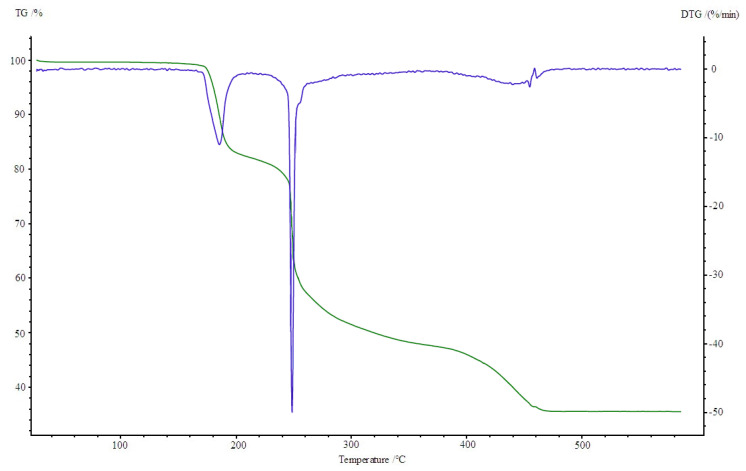
TG, DTG curves of Cu_2_(OAc)_4_(Im) complex in air.

**Figure 10:**
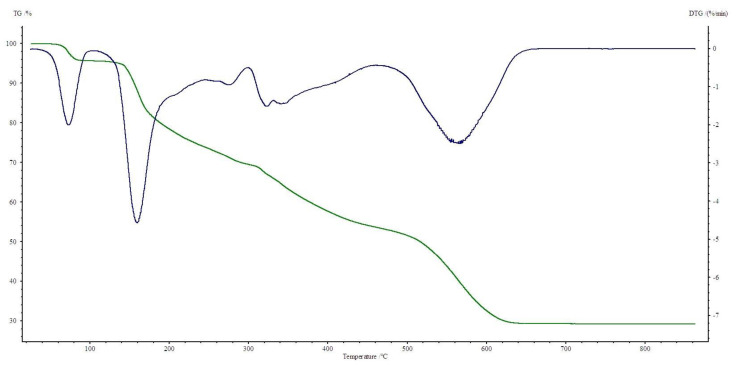
TG, DTG curves of Zn(OAc)_2_(Im)·H_2_O complex in air.

**Figure 11:**
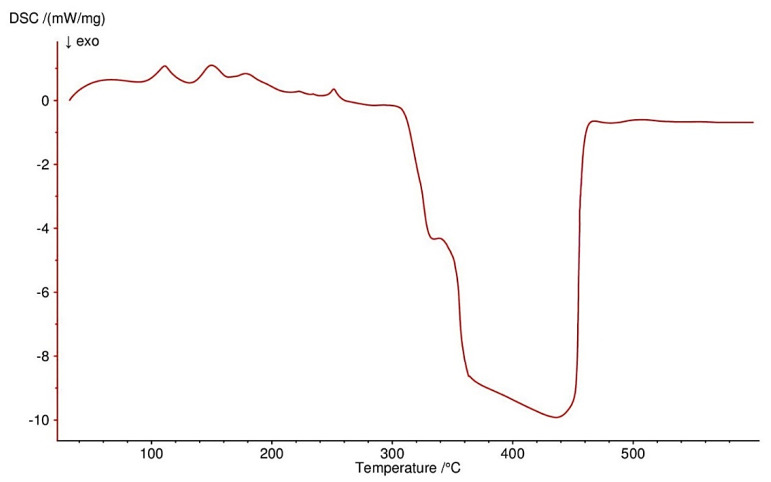
DSC curve of Co(OAc)_2_(Im)·H_2_O complex in air.

**Figure 12:**
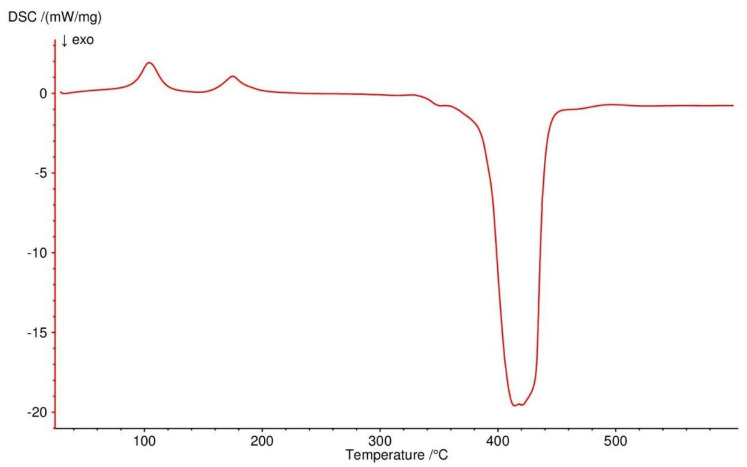
DSC curve of Ni(OAc)_2_(Im)_1.5_·2H_2_O complex in air.

**Figure 13:**
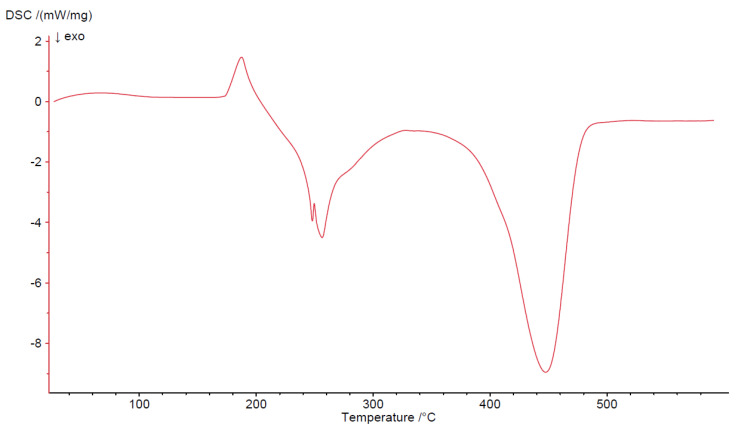
DSC curve of Cu_2_(OAc)_4_(Im) complex in air.

**Figure 14:**
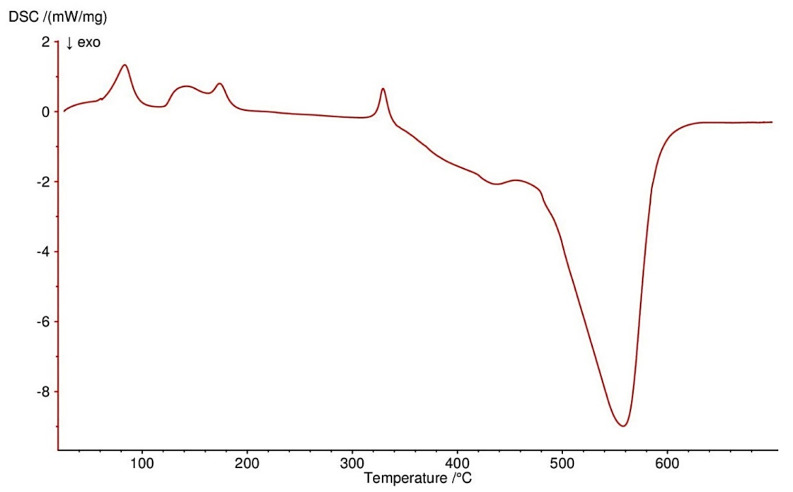
DSC curve of Zn(OAc)_2_(Im)·H_2_O complex in air.

**Table 1:** Analytical data for synthesized compounds.

No.	Compound	Analysis: (Found) Calculated/%			
		M	C	H	N
(I)	Co(OAc)_2_(Im)·H_2_O	22.40 (22.43)	31.95 (31.90)	4.56 (4.59)	10.64 (10.59)
(II)	Ni(OAc)_2_(Im)_1.5_·2H_2_O	18.64 (18.59)	32.39 (32.48)	5.08 (5.06)	13.34 (13.42)
(III)	Cu_2_(OAc)_4_(Im)	29.46 (28.94)	30.63 (31.18)	3.53 (3.50)	6.49 (6.45)
(IV)	Zn(OAc)_2_(Im)·H_2_O	24.25 (24.19)	31.15 (31.05)	4.45 (4.43)	10.39 (10.43)

**Table 2:** Fundamental FTIR bands [cm^−1^] for imidazole and -COO groups in obtained complexes.

Compound	ν(NH)	ν(CH)	ν(CN)	δ(NH), ν(CC), ν(CN)	*δ*(CH), *δ*(imidazole ring)	*π*(CH), *δ*(imidazole ring)	ν_as_(COO)	ν_s_(COO)
NaOAc	−	−	−	−	−	−	1578	1414
imidazole	3124	3014	1645	1540	937	894	−	−
Co(OAc)_2_(Im)·H_2_O	3139 3118	3058	1600	1546	952	883	1571	1415 1409
Ni(OAc)_2_(Im)_1.5_·2H_2_O	3120	3047	1612	1537	910	868	•	1415
Cu_2_(OAc)_4_(Im)	3143 3118	3056	1627	1552	916	869	1519	1409 1360
Zn(OAc)_2_(Im)·H_2_O	3139 3114	3058	1621 1610	1548	954	887	1577	1419

•—Overlaid by N-donor ligand

**Table 3:** Thermal decomposition data of obtained compounds.

Compound	Range of Decomposition/°C	DSC Peaks/°C	Mass Loss/%		Intermediate and Residue Solid Products
			Found	Calc.	
Co(OAc)_2_(Im)·H_2_O	80–125 125–225 225–410	110 endo 150, 180 endo 250 endo, 330–460 exo	4.0 17.0 49.5	3.43 16.35 49.72	Co(OAc)_2_(Im)·0.5H_2_O Co(OAc)_2_(Im)_0.5_ Co_3_O_4_
Ni(OAc)_2_(Im)_1.5_·2H_2_O	50–125 125–160 160–375 375–440	105 endo - 175 endo 350–440 exo	8.0 3.5 32.0 32.5	8.58 2.86 32.43 32.42	Ni(OAc)_2_(Im)_1.5_·0.5H_2_O Ni(OAc)_2_(Im)_1.5_ Ni(OAc)_2_ NiO
Cu_2_(OAc)_4_(Im)	165–200 200–340 340–490	190 endo 250, 260 exo 450 exo	16.0 34.0 13.5	15.78 34.23 13.12	Cu_2_(OAc)_4_ Cu_2_(OAc)_1.5_ CuO
Zn(OAc)_2_(Im)·H_2_O	50–100 125–310 310–650	80 endo 140, 170 endo 330 endo, 430–600 exo	4.0 28.0 37.5	3.34 28.62 37.86	Zn(OAc)_2_(Im)·0.5H_2_O Zn(OAc)_2_ ZnO
